# Sense of community and willingness to support malaria intervention programme in urban poor Accra, Ghana

**DOI:** 10.1186/s12936-018-2424-0

**Published:** 2018-08-10

**Authors:** D. Yaw Atiglo, Reuben Tete Larbi, Mawuli Komla Kushitor, Adriana A. E. Biney, Paapa Yaw Asante, Naa Dodua Dodoo, F. Nii-Amoo Dodoo

**Affiliations:** 1 Regional Institute for Population Studies (RIPS), University of Ghana, Legon, P. O. Box LG 96, Accra, Ghana; 2 Department of Psychology, University of Ghana, Legon, P. O. Box LG 84, Accra, Ghana; 3 Office of Research, Innovation and Development, University of Ghana, Legon, P. O. Box LG 571, Accra, Ghana; 4 The Pennsylvania State University, 211 Oswald Tower, University Park, PA 16802 USA

**Keywords:** Malaria, Urban poor, Sense of community, Willingness to participate, Contingent valuation experiment, Ghana

## Abstract

**Background:**

The extensive research on community members’ willingness to support malaria interventions ignores the role of psychosocial determinants. This study assesses the impact of individuals’ sense of community (perceptions of community cohesion, altruism, seeking help from neighbours and migrant status) on their willingness to participate in a mosquito control programme using data on 768 individuals from the 2013 RIPS Urban Health and Poverty Survey in poor coastal communities in Accra, Ghana. A contingent valuation experiment was employed to elicit individuals’ willingness to support the programme by contributing nothing, labour time/money only or both.

**Results:**

Findings show that different dimensions of sense of community related differently with willingness to support the programme. Perceived community cohesion was associated with lower odds while help-seeking from neighbours and being a migrant were associated with higher odds of supporting the programme. Altruism was the only dimension not linked to willingness to participate.

**Conclusions:**

Different dimensions of sense of community are associated with community members’ willingness to provide labour, time or both to support the malaria eradication programme. The findings of this study have implications for targeting social relational aspects, in addition to geographical aspects, of communities with malaria-resilient policy and intervention. They also warrant further research on psychosocial factors that predict willingness to support health programmes in urban poor settings.

## Background

Malaria is a parasitic disease epidemic that recurrently confronts populations in sub-Saharan Africa. The economic, health and social impacts of malaria incidence in low and middle-income economies are dire and well-documented. The Intergovernmental Panel on Climate Change (IPCC), under its high confidence projections, indicates an increase in climate-related infectious diseases due to rising temperatures and rainfall variability (IPCC [1]). This means that more adaptive measures will be required in response to the rising risk of malaria transmission. Transmission of the disease can be suppressed through various vector control measures including the use of insecticide-treated nets (ITN), indoor residual spraying (IRS) and larvicides. ITNs and indoor spraying techniques are most effective against indoor transmission only but largely ineffective against outdoor transmission [2–6]. Other widely used measures include treatment-seeking behaviours which are less effective than vector control strategies towards eradicating the epidemic [5]. Vector source control potentially complements various strategies for adult mosquito control, especially in urban areas [4]. The use of larvicides, however, is less common and very often dismissed though it potentially fits into integrated vector management [2, 3, 5, 7]. High-efficacy larval control has been shown to have an effective controlling outcome on mosquito-transmitted infections [3, 4, 7, 8], although community knowledge of larval source control is inadequate, particularly in sub-Saharan Africa [2, 7].

Building resilient cities, as a major global development agenda, emphasizes the ability of communities to self-organize resources in building capacity to respond to stressors. Though governments and multilateral donors may fund large-scale malaria control interventions, such as the roll-back malaria programme, sustainability is assured when community members take responsibility and ownership of such programmes [9–12]. Social resilience to diseases through robust health systems, access to resources and community agency, provides communities adequate capacity to respond to epidemics [13]. In providing malaria interventions, equity is key as not everyone in a community can afford or sustainably implement household based malaria control. Community members may demonstrate altruistic willingness to pay for intervention schemes which eventually benefit the poor in those communities [11, 14].

Assessments of community members’ willingness to pay for malaria prevention and treatment abound relative to other tropical illnesses [15, 16]; however, studies overlook the importance of psychosocial factors aside the demographic, economic, malaria-related and environmental factors. These studies also give more prominence to personal and household protection measures, such as the use of ITNs and IRS, to the neglect of community-level outdoor measures such as larval control [5, 17–20]. Further, existing studies on the altruistic willingness to support malaria intervention tend to focus more on ITN distribution and uptake in rural areas [14, 17]. Very little is known about willingness to support malaria prevention programmes in urban areas, much less among the urban poor. Meanwhile, urban health contributes to the public health burden in developing countries, particularly in rapidly urbanizing sub-Saharan African countries where high rates of communicable diseases persist [21].

This current study proffers an additional dimension which assesses the sense of community belonging and individuals’ altruistic dispositions towards their willingness to support community-led vector source control intervention programmes among the urban poor. The extent to which individuals’ altruism and sense of community predict their willingness to support a larval control intervention with time and/or money was assessed in three poor urban communities in Ghana. This is a significant contribution towards the attainment of the sustainable development goal (SDG) 3 on good health and well-being and SDG 11 on making “cities and human settlements inclusive, safe, resilient and sustainable” [22].

### Understanding sense of community and the willingness to participate in a community-based intervention in the urban poor context

Urban populations generally have lower malaria burden than their rural counterparts due to the availability of preventative and curative services [23]. That notwithstanding, poor urban and slum communities lack access to health care, making them more vulnerable to malaria [24]. Slum communities are also noted to have a high sense of social mistrust, disorder, poor quality housing and poor sanitation infrastructure among others. Poor sanitation and drainage amidst dense population concentrations expose growing urban slum residents to the risk of malaria, diarrhoea and other related communicable diseases. Despite this, poor urban communities have the potential for “communal self-organization and clever construction” if they come together and want to change their circumstances [25].

As indicated by the extant literature, people’s socioeconomic and demographic characteristics also bear significant association with attitude towards community self-help initiatives. Knowledge about and ability to prevent and treat malaria also influence control measures that people adopt [26]. These are key indicators of community participation in malaria interventions; however, psychosocial factors, including how community members perceive their community or their ‘sense of community’ are lacking in the discourse on malaria and general health interventions. Sense of community is positively associated with active community participation [27–30] and both enhance social development and reinforce social capital towards community development [29]. In spite of the challenges associated with community-based intervention programmes, they have been apprised as successful by empowering community members and making them feel responsible as owners [5, 20, 28].

Sense of community is a multidimensional construct, comprising membership, influence, shared emotional connections and fulfilment of needs, and interpersonal connections [29]. This four-dimensional model was adopted in conceptualizing sense of community among urban poor residents. Perception of social cohesion and willingness to ask for help indicates fulfilment of needs; altruism indicates influence, and migrant status indicates both membership and shared emotional connections. These socio-psychological processes impact community participation in health interventions in urban poor communities in Ghana. Even though a community may imply both geographical and social relational units, the latter reflects common interest and bonding among people [27, 28]. Community-based health programmes are more likely to be successful when members have a high sense of community belongingness and emotional connectedness rather than being mere residents of a geographical location [28]. For an intervention that requires community participation, focusing on specific geographical location and neglecting people’s sense of community may fail to yield adequate community mobilization. Given that the use of microbial larvicides is not a familiar exercise in the study communities, it was envisaged that willingness to participate is likely to be influenced more by respondents’ sense of community.

## Methods

### Study setting

The study was conducted in three densely populated but resource-constrained coastal urban communities in Accra: James Town, Ussher Town and Agbogbloshie. Agbogbloshie, relative to James Town and Ussher Town, shows more slum characteristics and is more prone to flooding and malaria risk due to its proximity to the Korle Lagoon and poor drainage system, with a major channel draining Accra passing through it [21, 31]. James Town and Ussher Town, spatially close to each other, constitute the Ga-Mashie Traditional Area and are largely inhabited by indigenous Ga people whereas Agbogbloshie is typically a migrant slum and market area though some residents trace their roots to as far back as the 1960s. James Town exhibits the highest socioeconomic standards and has a significantly fair balance of indigenous and migrant populations [31]. All three communities form part of the Ashiedu-Keteke sub-metro area in the Accra Metropolitan Area (AMA). Poor sanitation and drainage systems in the AMA make the area liable to flooding and breeding of mosquitoes, especially during the rainy season [32]. Detailed information on the study area can be found elsewhere [21, 31, 32].

### Questionnaire and data collection

This paper utilizes data on 768 individuals from the third wave of the Urban Health and Poverty Survey, conducted by the Regional Institute for Population Studies (RIPS) of the University of Ghana between September and October, 2013. A systematic sampling approach was employed to select households from 29 enumeration areas (EAs) sampled proportionally to the sizes of the three localities in the study area. Two sets of questionnaires, a household questionnaire and an individual questionnaire, were presented to households. The household questionnaire was presented to the household head, and in their absence an adult representative was interviewed. All women aged 15–49 and men aged 15–59 in a household were eligible to respond to the individual questionnaire. The interviews were mainly conducted in English, Ga and Twi though there were a few instances where interviews were conducted in Hausa or Ewe according to the preferred choice of the respondent. The interviews were conducted by trained interviewers through house visits. This study uses data from both the household and individual questionnaires.

The study protocol was reviewed and received ethical approval from the Institutional Review Board of the Noguchi Memorial Institute for Medical Research (NMIMR-IRB) in Ghana in August 2013. In addition, the survey team received approval from the community leaders including chiefs, opinion leaders and assembly members before the study commenced at each phase. Each respondent consented to participate in the study by signing or with a thumb print after being given information about the research and assured of confidentiality before the interview was conducted.

### Contingent valuation experiment

A hypothetical scenario was presented to detail what the larviciding programme entailed; the benefits, limitations and prospects of the programme. Detailed information on the intervention programme were presented as follows. The Ministry of Health would introduce Fenthion (82.5% w/v), a larvicide compound with long residual effect to be used in stagnant polluted non-drinking water for animals or humans. The chemical (larvicide) would be applied once a week and would reduce the incidence of malaria by up to 20%. Application would be revised based on preliminary observations and residual effect of the compound. Costs of the initiative, including chemical, water, sprayer and labour time could not be borne by government budget. The community would have to support the initiative by contributing labour time or money or both labour time and money. Participants were then asked if they would be willing to contribute money or time or both and if so how much time (in hours) or money (in Ghana cedis) they were willing to contribute.

The authors acknowledge the limitations of open-ended contingent valuation method, that survey respondents may desirably exaggerate willingness to support than they would if they were directly primed to it [33]. The method is however justifiable and valuable for assessing people’s valuation of goods, services and programmes, particularly when it includes both monetary and labour contributions [17, 34].

### Measures

*Dependent variable* The outcome variable was willingness to support, measured as ‘not willing’, ‘willing to contribute money only or time only’, and ‘willing to contribute both money and time’.

*Independent variables* The four main independent variables are jointly referred to as individuals’ sense of community. Sense of community variables included ‘perception of community cohesion’, ‘migrant status/duration of residence’, ‘altruism (likelihood to offer help to neighbours)’ and ‘likelihood to ask neighbours for support’.

*Perception of community cohesion* is a scale measured by six items that ask the extent to which respondents agree with each of the following six statements: *(a) this is a close*-*knit community; (b) people in this community are willing to help each other; (c) people in this community can be trusted; (d) people in this community watch out for each other; (e) people in this community would work together if there was a serious problem; (f) people in this community look out mainly for the welfare of their families and they are not much concerned with community welfare*. Responses were coded as 1—strongly disagree to 4—strongly agree. The sixth statement which suggests more self-centredness than community cohesion was reverse-coded.

The *self-reported altruism* scale is made up of five statements that measure individuals’ likelihood to help a neighbour who needed: *(a) to borrow money; (b) a small amount of food; (c) somewhere to spend the night; (d) medicine or medical care; (e) to talk about something worrying them*. *Likelihood to request help* is constructed on a similar scale with questions that elicit individuals’ attitude towards willingness to ask their neighbours for help when they needed the same items mentioned for the altruism scale above. Both the altruism scale and likelihood to request help scales involved codes as 1—never 2—not likely 3—somewhat likely 4—very likely.

*Migrant status* is measured by duration of residence in community. Three categories of residents are (i) non-migrants (lived in the community since birth); (ii) recent migrants (moved into community less than 5 years ago); and (iii) longer-staying migrants (moved into community five or more years ago).

*Control variables* The control variables were selected based on their association with willingness or capacity to support malaria intervention programmes and also through their risk for malaria. These included spatial variables (e.g. distance from drain), sociodemographic and malaria-related (knowledge and experience) variables. The main environmental variables included the *distance from open drain* to where the respondent resides and *locality of residence*. The distinct spatial and demographic features of these localities make us classify them under environmental characteristics. Sociodemographic characteristics controlled for in the models include *age*, *sex*, *income*, *educational attainment* and *ethnicity*.

Malaria-specific variables include *knowledge of malaria transmission*, *individual’s last malaria episode (history)*, *perceived risk of malaria*, *perception of community malaria incidence*, *household capacity to prevent or treat malaria*, and *knowledge of malaria casualty*. Knowledge of malaria transmission was derived from erratic notions about malaria causes including drinking dirty water, witchcraft or standing water.

### Data analysis

The individual adult community member was the unit of analysis. The frequency distribution and summary of categorical and continuous variables, respectively, are presented in the first part of the analysis. Bivariate analyses involved Chi square tests of associations and one-way ANOVA. The multivariate analyses include two multinomial logistic regression models. The first model includes only the sense of community variables while the second model controls for environmental, sociodemographic and selected malaria-related characteristics of respondents. The malaria-related variables for the final model were based on their significant association with willingness to participate at the bivariate level. Given that the outcome of the model was a multi-category nominal variable [35], the multinomial logistic regression model was fitted according to the equation:

1 $$\ln \frac{p\left( x \right)}{1 - p\left( x \right)} = \beta 0 + \beta 1x,$$

where $p$ is the probability that an event (willingness to contribute) occurs, and $p \frac{p\left( x \right)}{1 - p\left( x \right)}$ is the odds ratio.

The odds of the event (willingness to contribute) associated with y = 1 [35], when participants are willing to contribute money or time only or both money and time is given as

2 $$\frac{p\left( x \right)}{1 - p\left( x \right)} = e^{\beta 0 + \beta x} = e^{\beta 0} (e^{\beta 1} )^{x} .$$

This means that a change in predictor variable *x* results in the odds of the specified outcome event (willingness to contribute) being multiplied by $e^{\beta 1}$. It implies that when $\beta_{1} = 0, e^{{\beta_{1} }} = 1$ and the odds of the event does not change when the value of the explanatory variable changes. Hence the odds ratio of a person’s willingness to contribute money or time or both when the explanatory variable has value of $x$ to $x + 1$ is $e^{\beta 1}$.

Model fit tests were performed to ensure that the variables which were incorporated based on theory were not overfitting the model due to the small sample size. First of all, the “fitstat” STATA command was used to compare the final model with other models which included combinations of control variables. This command generates statistics which include the pseudo R squared, count R squared, McFadden’s R squared, AIC, BIC among others. Results of the model are displayed with lower AIC and BIC levels, as these indicate the predictive ability of models. The lower the values the more predictable the model is for fitting new data though these could imply lower Pseudo R squared values as well. The AIC and BIC were preferred over the R squared because including predictor variables that are not even theoretically correlated may produce higher R squared values.

## Results

### Univariate results

Results of univariate analyses are presented in Tables 1 and 2. Table 1 describes key nominal variables included in the analysis and demographic characteristics of study respondents. The analysis involved 768 participants. A small proportion (10.9%) of respondents stated that they would contribute neither time nor money, yet, close to half of all the respondents indicated willingness to contribute to both money and time (46.7%) to the proposed community-based malaria intervention. Majority of the respondents were from Ussher Town (60%) while the lowest proportion was from Agbogbloshie. An important spatial measure included in the analysis is the distance to open drains. More than 80% of the respondents reported living within 50 m of an open drain.

**Table 1:** Percent distribution of respondents and willingness to contribute by socio-demographic variables

Categorical variables	Not willing	Money only or time only	Both	Total
	Freq. (%)	Freq. (%)	Freq. (%)	Freq. (%)
*Years lived in community*	**χ**^**2**^ ** = ** **10.60**		**Pr = ** **0.031**	
5 years and below	7 (7.07)	42 (42.42)	50 (50.51)	99 (100.00)
> 5 years	16 (16.00)	108 (45.57)	113 (47.68)	237 (100.00)
Since birth	61 (14.12)	175 (40.51)	196 (45.37)	432 (100.00)
Total	84 (10.94)	325 (42.32)	359 (46.74)	768 (100.00)
*Knowledge of malaria transmission*	**χ**^**2**^ **=** **2.8897**		**Pr** **=** **0.576**	
Low	14 (9.59)	66 (45.21)	66 (45.21)	146 (100.00)
Moderate	45 (11.66)	168 (43.52)	173 (44.82)	386 (100.00)
High	25 (10.59)	91 (38.56)	120 (50.85)	236 (100.00)
Total	84 (10.94)	325 (42.32)	359 (46.74)	768 (100.00)
*Last malaria episode*	**χ**^**2**^ **=** **1.3698**		**Pr** **=** **0.729**	
1 month or less	27 (13.11)	87 (42.23)	92 (44.66)	206 (100.0)
Over 1 month ago	57 (10.23)	236 (42.37)	264 (47.40)	557 (100.0)
Total	84 (10.94)	323 (42.32)	356 (46.74)	763 (100.00)
*Perceived risk of malaria*	**χ**^**2**^ **=** **0.6314**		**Pr** **=** **0.729**	
No/low risk	40 (10.18)	164 (41.73)	189 (48.09)	393 (100.00)
Moderate/high risk	42 (11.45)	158 (43.05)	167 (45.50)	367 (100.00)
Total	82 (10.79)	322 (42.37)	356 (46.84)	760 (100.00)
*Know someone died of malaria*	**χ**^**2**^ **=** **8.3696**		**Pr** **=** **0.015**	
Yes	5 (4.24)	47 (39.83)	66 (55.93)	118 (100.00)
No	78 (12.15)	274 (42.68)	290 (45.17)	642 (100.00)
Total	83 (10.92)	321 (42.24)	356 (46.84)	760 (100.00)
*Capacity to prevent/treat*	**χ**^**2**^ **=** **3.4371**		**Pr** **=** **0.179**	
Inadequate capacity	27 (10.55)	119 (46.48)	110 (42.97)	256 (100.00)
Adequate capacity	56 (11.13)	199 (39.56)	248 (49.30)	503 (100.00)
Total	83 (10.94)	318 (41.90)	358 (47.17)	759 (100.00)
*Perceived malaria incidence*	**χ**^**2**^ **=** **10.0486**		**Pr** **=** **0.040**	
Increasing	13 (6.63)	77 (39.29)	106 (54.08)	196 (100.00)
Declining	49 (12.25)	182 (45.5)	169 (42.25)	400 (100.00)
Remains the same	21 (13.04)	65 (40.37)	75 (46.58)	161 (100.00)
Total	83 (10.96)	324 (42.80)	350 (46.24)	757 (100.00)
*Distance to main drain* (m)	**χ**^**2**^ **=** **12.0792**		**Pr** **=** **0.017**	
< 10	24 (7.92)	128 (42.24)	151 (49.83)	303 (100.00)
10–50	49 (14.12)	134 (38.62)	164 (47.26)	347 (100.00)
> 50	11 (9.57)	60 (52.17)	44 (38.26)	115 (100.00)
Total	84 (10.98)	322 (42.09)	359 (46.93)	765 (100.00)
*Locality of residence*	**χ**^**2**^ **=** **13.4637**		**Pr** **=** **0.009**	
Agbogbloshie	11 (10.68)	54 (52.43)	38 (36.89)	103 (100.00)
James Town	20 (9.26)	105 (48.61)	91 (42.13)	216 (100.00)
Ussher Town	53 (11.80)	166 (36.97)	230 (51.22)	449 (100.00)
Total	84 (10.94)	325 (42.32)	359 (46.74)	768 (100.00)
*Sex*	**χ**^**2**^ **=** **0.6659**		**Pr** **=** **0.717**	
Male	40 (11.98)	139 (41.62)	155 (46.41)	334 (100.00)
Female	44 (10.14)	186 (42.86)	204 (47.00)	434 (100.00)
Total	84 (10.94)	325 (42.32)	359 (46.74)	768 (100.00)
*Educational attainment*	**χ**^**2**^ **=** **3.3941**		**Pr** **=** **0.494**	
None/prim	15 (8.24)	81 (44.51)	86 (47.25)	182 (100.00)
Junior high/middle school	40 (12.31)	141 (43.38)	144 (44.31)	325 (100.00)
Senior High School (SHS)/Higher	29 (11.11)	103 (39.46)	129 (49.43)	261 (100.00)
Total	84 (10.94)	325 (42.32)	359 (46.74)	768 (100.00)
*Ethnicity*	**χ**^**2**^ **=** **3.3003**		**Pr** **=** **0.509**	
Ga	53 (11.42)	189 (40.73)	222 (47.84)	464 (100.00)
Akan	15 (8.2)	82 (44.81)	86 (46.99)	183 (100.00)
Others	16 (13.22)	54 (44.63)	51 (42.15)	121 (100.00)
Total	84 (10.94)	325 (42.32)	359 (46.74)	768 (100.00)

*Pr* p-values, *Freq.* frequency

**Table 2:** Summary statistics of respondents indicating distribution of participants by willingness to support larvicides intervention using one-way ANOVA

Continuous variables	Mean (SD)	Mean of respondents not willing to contribute (SD)	Mean of respondents willing to contribute money or time only (SD)	Mean of respondents willing to contribute both time and money (SD)	F-statistic
*Community characteristics*					
Perception of community cohesion (6–24)	15.565 (3.121)	16.309 (2.974)	15.465 (2.939)	15.488 (3.298)	2.67^^^
Willingness to request help score (5–20)	10.723 (4.623)	8.798 (3.620)	11.348 (4.670)	10.609 (4.667)	10.62***
Altruism score (5–20)	15.570 (4.088)	14.440 (4.860)	15.606 (3.933)	15.802 (3.998)	3.82*
*Sociodemographic characteristics*					
Age	30.350 (10.830)	29.667 (9.996)	30.317 (10.923)	30.540 (10.955)	0.15
Monthly income	190.0635	161.369 (187.349)	193 (250.545)	193.336 (253.213)	0.64

*SD* standard deviation

^^^p < 0.10, * p < 0.05, ** p < 0.01, *** p < 0.001

Females outnumbered males in the study. Reflective of national patterns, more people had attained junior high/middle school than secondary and tertiary education together while a little under a quarter (23.7%) had no formal education or had received only primary education. About half the respondents perceived they had moderate to high risk of malaria infection and over half (57.4%) perceived they had adequate capacity to treat malaria on their own. Over 80% of the respondents had adequate to good knowledge of malaria. Furthermore, under a fifth of community members (15.5%) knew someone who had died of malaria making it an existential threat. In addition, three quarters of the sample perceived that the burden of malaria had either remained the same or was decreasing.

The perception of community cohesion score is generally high among respondents with a mean score of 15.6. Willingness to request help is much lower than altruism scores with means of 10.7 and 15.6 respectively. The mean age of respondents was 30 years, specifically 31.6 years for males and 29.2 years for females. Mean monthly income was 190 Ghana cedis.^1^

### Bivariate results

The analysis of variance results showed that neither income nor age of an individual had a significantly different effect on their willingness to contribute (p > 0.05). Mean score of willingness to request help was highest among respondents willing to contribute only time or only money and lowest among those not willing to contribute. The altruism score was highest among those willing to contribute both time and money but lowest among those not willing to support. Higher proportions of all categories of residents were willing to contribute both time and money, particularly recent migrants (50.5%).

Further, a person’s willingness to contribute to the community malaria intervention was associated with the years lived in the community (χ^2^ = 10.60, p < 0.05), knowing someone who died of malaria in the past 12 months (χ^2^ = 8.37, p < 0.05), perceived malaria incidence (χ^2^ = 10.05, p < 0.05), distance to main drain (χ^2^ = 12.08, p < 0.05), and locality of residence (χ^2^ = 13.46, p < 0.001).

Particularly, higher proportions of people who knew someone who died of malaria 12 months before the survey (55.9%) were willing to contribute both time and money than their counterparts who did not know anyone who died of malaria (45.17%). Concerning perceived malaria incidence, more than half (54.08%) of those who perceived an increasing incidence were willing to contribute both time and money compared with those who perceived a decreasing incidence (42.25%). Respondents who stayed within 10 m from the main drain formed the highest proportion (49.83%) of those willing to contribute both time and money; a little more than a third (38.26%) of those who reside 50 m or more from the main drain were willing to contribute both time and money. However, about one of every two respondents (52.17%) who stay over 50 m from the drain were willing to contribute either money or time. The highest proportion of individuals willing to contribute both money and time resided in Ussher Town and the highest proportion who were willing to contribute either money or time resided in James Town.

Other variables included in the study but which did not have any significant association with willingness to contribute towards the malaria intervention are educational attainment, sex of respondent, knowledge of malaria transmission, ethnicity, and the perceived personal capacity to prevent or treat malaria.

### Multivariate analysis

In Table 3—Model 1, different sense of community indicators showed different associations with willingness to contribute time or money or both. A unit increase in perceived community cohesion score was associated with lower odds of willingness to contribute time or money or both relative to non-willingness. Willingness to request help from neighbours was positively correlated with the likelihood of willingness to support with either time or money only (1.16 times) or both (1.10 times) than no willingness to support. Altruism, measured as readiness to give assistance to neighbours when they need them, showed no significant relationship with willingness to support. Longer staying migrants were more than twice as much as non-migrants to be willing to support. A similar risk ratio is observed for recent migrants though the statistical difference between non-migrants and recent migrants was a modest one.

**Table 3:** Multinomial logistic regression results showing odds of willingness to contribute time or money only or both time and money relative to non-willingness to contribute by individuals’ sense of community, environmental, malaria-related and sociodemographic characteristics

Categorical variable	Model 1 (pseudo R^2^ = 0.031; N = 766; χ^2^ = 45.53; prob > χ^2^ = 0.0000)				Model 2 (pseudo R^2^ = 0.071; N = 746; χ^2^ = 102.53; prob > χ^2^ = 0.0000)			
	Money or time only		Both money and time		Money or time only		Both money and time	
	RRR (SE)	(95% CI)	RRR (SE)	(95% CI)	RRR (SE)	(95% CI)	RRR (SE)	(95% CI)
*Sense of community variables*								
Duration of residence in community								
Non-migrant *ref*								
Recent migrant (5 years or less)	2.117 (0.936)^^^	(0.889, 5.037)	2.193 (0.955)^^^	(0.934, 5.149)	2.424 (1.209)^^^	(0.912, 6.444)	2.843 (1.401)*	(1.082, 7.471)
Longer-staying migrant (over 5 years)	2.277 (0.716)**	(1.229, 4.218)	2.058 (0.640)*	(1.119, 3.787)	2.941 (1.095)**	(1.418, 6.100)	2.938 (1.080)**	(1.429, 6.040)
Perception of community cohesion	0.889 (0.037)**	(0.819, 0.965)	0.899 (0.037)**	(0.829, 0.974)	0.836 (0.039)***	(0.761, 0.916)	0.857 (0.040)**	(0.783, 0.938)
Willingness to request help score	1.156 (0.038)***	(1.084, 1.232)	1.105 (0.035)**	(1.038, 1.177)	1.181 (0.042)***	(1.102, 1.266)	1.121 (0.039)**	(1.047, 1.200)
Altruism score	1.015 (0.038)	(0.956, 1.077)	1.045 (0.031)	(0.986, 1.108)	1.011 (0.033)	(0.948, 1.079)	1.035 (0.033)	(0.971, 1.102)
*Environmental characteristics*								
Distance from open drain								
Less than 10 m *ref*								
10–50 m					0.436 (0.132)**	(0.241, 0.790)	0.470 (0.139)*	(0.262, 0.840)
Over 50 m					1.507 (0.670)	(0.630, 3.602)	0.997 (0.444)	(0.416, 2.388)
Locality of residence								
Agbogbloshie *ref*								
James Town					2.673 (1.370)^	(0.979, 7.299)	3.703 (1.923)*	(1.338, 10.247)
Ussher Town					1.515 (0.733)	(0.587, 3.910)	3.305 (1.614)*	(1.269, 8.605)
*Malaria*-*related characteristics*								
Perceived prevalence of malaria								
Increasing *ref*								
Decreasing					0.875 (0.324)	(0.424, 1.806)	0.592 (0.214)	(0.292, 1.204)
Remained same					0.505 (0.211)	(0.223, 1.144)	0.421 (0.171)*	(0.190, 0.933)
Personal knowledge of malaria death								
Yes					1.952 (1.003)	(0.713, 5.346)	2.529 (1.277)^	(0.940, 6.806)
No *ref*								
*Sociodemographic characteristics*								
Gender								
Male *ref*								
Female					1.165 (0.319)	(0.682, 1.992)	1.104 (0.297)	(0.651, 1.870)
Age					0.995 (0.014)	(0.969, 1.023)	0.998 (0.014)	(0.972, 1.025)
Monthly income					1.001 (0.001)	(0.999, 1.002)	1.001 (0.001)	(0.999, 1.002)
Educational attainment								
None/primary *ref*								
JHS/middle					0.613 (0.221)	(0.303, 1.242)	0.691 (0.247)	(0.343, 1.394)
SHS/higher					0.717 (0.284)	(0.330, 1.560)	0.806 (0.316)	(0.374, 1.738)
Ethnicity								
Ga *ref*								
Akan					1.329 (0.515)	(0.621, 2.842)	1.329 (0.515)	(0.621, 2.842)
Others					0.923 (0.359)	(0.430, 1.978)	0.923 (0.359)	(0.430, 1.978)
Constant	3.518 (2.739)	(0.765, 16.178)	3.488 (2.684)	(0.772, 15.761)	8.013 (10.097)	(0.678, 94.701)	4.866 (6.075)	(0.421, 56.201)

*RRR* relative risk ratios, *SE* standard error

^ p < 0.10, * p < 0.05, ** p < 0.01, *** p < 0.001

In Model 2, where environmental, sociodemographic and selected malaria-related characteristics were controlled for, the same patterns were observed but the statistical powers were improved for all sense of community indicators. Longer staying migrants were more than twice as likely to be willing than not willing to support compared with the non-migrants. In this model, recent migrants were significantly more likely (2.4 times) than non-migrants to be willing to give time or money only than the non-migrants. Likelihood of willingness to give both time and money was increased for both categories of migrants when all other variables were controlled for.

Environmental characteristics had some association with willingness to support. Those who lived about 10–50 m away from open drain were less likely (0.47 times) than those living within a 10 m radius of open drains to be willing to contribute both time and money. Similarly, living 10–50 m away from open drain was associated with lower likelihood of willingness to contribute time only or money only. Residence in James Town and Ussher Town was associated with higher likelihood of willingness (3.7 and 3.3 times respectively) to contribute both time and money compared with residence in Agbogbloshie.

Interestingly, of the two selected malaria-related variables, significant at the bivariate level, only perceived prevalence of malaria was significantly associated with willingness to contribute both time and money but not time or money only. Also, none of the background sociodemographic and economic variables controlled for in Model 2 (age, gender, education, ethnicity and income) was significantly associated with willingness to support the intervention.

## Discussion

Malaria is a major cause of morbidity and mortality in SSA. Efforts to build malaria-resilient communities should ensure inclusive participation of community members for a higher sense of community ownership and sustainability. Sense of community facilitates community participation [29], therefore, this framework was employed to understand urban poor communities’ willingness to participate in self-help programmes. More specifically, the study sought to investigate the relationship between people’s sense of community and their willingness to contribute either money or labour time or both to a malaria control intervention in three malaria-endemic coastal urban poor communities in Accra, Ghana. Sense of community has not been considered in earlier studies that have investigated the determinants of community members’ willingness to contribute towards a malaria programme. The results revealed that an individual’s sense of belongingness to the community, their migration status, and environmental factors such as their proximity to a drain and locality of residence explained their willingness or otherwise to contribute to the intervention.

The four dimensions of sense of community are differently correlated with willingness to participate in the malaria intervention programme. First, although community cohesion is generally expected to benefit community members in terms of reciprocity and community assistance in closely knit communities, the findings suggest that a higher perception of community cohesion is associated with lower odds of willingness to contribute time or money or both to the community-based malaria intervention programme. While this observation is not entirely expected, it is not surprising. There is ample evidence to show that the effect of social cohesion on community reciprocity is mixed [21, 36, 37]. As some studies have suggested that in closely knit communities a higher sense of social cohesion is associated with higher altruistic tendencies [38], there is also evidence that suspicion is higher in such communities [39]. A sense of suspicion amongst community members may limit the willingness of people to contribute to a malaria intervention even though the intention is beneficial. Thus, community cohesion does not necessarily translate into social capital for community members as it was found that respondents’ perception of the presence of a close-knit community is rather inhibiting of their willingness to contribute time or money to the intervention programme. This could also explain why altruism, defined as an individual’s disposition towards willingness to help other community members, lacks a statistically significant association with willingness to contribute either time or money to the intervention programme. Notably, however, willingness to request assistance from neighbours is positively associated with willingness to contribute money and/or time to the intervention programme. This relationship may signify that individuals who can trust other community members for assistance may be more willing to contribute to community intervention programmes.

The study also observed that locality and migration status were significant predictors of the willingness to contribute to money or time or both. Residents in James Town and Ussher Town had a higher likelihood of willingness to contribute to time or money or both than residents in Agbogbloshie. This finding may be explained by the composition of the three study communities. First, even though all three communities have large populations of migrants, the purpose and type of migration is different for Agbogbloshie and Ga-Mashie (James Town and Ussher Town). Migration in Agbogbloshie is transient; a huge market situated in the community attracts scores of migrants from all over the country [32]. The purpose of their migration is to transact business. A lot of these respondents may not have the intention of staying permanently in the community and, therefore, may be less willing to contribute to a community based intervention programme. In addition to that, these migrants may not identify strongly with the community and will not commit to a long-term community intervention effort. The needs of the Agbogbloshie community are therefore different from those of James Town and Ussher Town where both non-migrants and migrants have a higher commitment to the community projects. In comparing migrants to non-migrants however, migrants who had stayed longer in the community were associated with a higher commitment to either time or money or both.

Further, studies show that in resource-scarce environments, health interventions are not the priority of community members as they are more concerned with pressing basic needs such as food, water and shelter. Health is only prioritised when a disease condition necessitates it, such as an acute condition that requires immediate medical attention. Hence, a malaria intervention that is focused on preventing the occurrence of malaria in the community may therefore not receive a lot of support. Further, the lack of willingness to support a community based intervention that is focused on disease prevention may be at odds with a national attitude that is more curative than preventative. There is also a lot of evidence to show that for malaria, self-treatment is the dominant health behaviour associated with its treatment [40, 41, 42]. Community members perhaps feel that malaria treatment should be managed at the individual level or the household level and therefore should not require a community effort. In addition, there are several community-based self-treatment strategies widely available and shared amongst community members such that people may not accept the need for a malaria intervention [42]. Put simply, malaria is probably not acknowledged as an important need in the community to warrant a combined community effort.

The findings of the study will be useful for research, policy and practice towards building malaria-resilient communities. Contribution towards a community health intervention driven by a sense of community is crucial for labour and monetary resource mobilization. The key to building a resilient community involves targeting geographical as well as relational dimensions of community belongingness. This is demonstrated in the significant association between both sense of community and locality of residence and the willingness to contribute money or time or both towards the community programme. The other principal factor that was beyond the scope of this study is the existence of community-level institutions such as chieftaincy, self-help groups, and youth groups that provide human resource and also drive other resources of resilience.

Also, it is important to evaluate the content of instruments used to assess willingness to support an intervention. A horizontal approach to community participation which involves facilitating communities to identify their problems and suggest solutions is more likely to enhance willingness of community members than the vertical approach where programme objectives are developed at a central point and deployed to engage communities [43]. The former is more likely to empower community members to identify with the project and participate to ensure its sustainability [43]. The contingent valuation experiment in this study presents a vertical participatory approach. This may account for the relationships observed that higher sense of community cohesion is associated with lower willingness to support and the lack of association with altruistic disposition.

## Conclusions

This study sought to establish the association between individuals’ sense of community and willingness to support an intended malaria intervention with labour time or money or both in urban poor communities in Accra, Ghana. The study findings show that among the urban poor, some dimensions of individuals’ sense of community are associated with their willingness to support the programme. Other environmental characteristics and malaria-related knowledge or experience influence individuals’ willingness to support malaria intervention programmes. Willingness to participate towards community health development goes beyond individuals’ residential environments and resource availability into other social relations that engender a sense of community.

## Footnotes

1 One Ghana cedi at the time was about 0.467 US dollars.
